# A peculiar appendix: A case report

**DOI:** 10.1016/j.ijscr.2022.107726

**Published:** 2022-10-10

**Authors:** B.M. Munasinghe, C.T. Karunatileke, G.G.C. Hewawasam, C.G. Hewavitharane, Karl Kuruppu

**Affiliations:** a Department of Anaesthesiology and Intensive care, District General Hospital, Mannar, Sri Lanka; b Department of Surgery, District General Hospital, Mannar, Sri Lanka; c Department of Anaesthesiology and Intensive care, Queen Elizabeth the Queen Mother Hospital, Ramsgate Rd, Margate, UK

**Keywords:** Appendix, Ascending colon, Appendicular agenesis, Appendicular duplication, Case report

## Abstract

**Introduction and importance:**

Acute appendicitis is a clinical diagnosis with marked variations in the clinical presentation, the latter resultant of varied anatomical positions of the appendix.

**Presentation of case:**

Here we present the first documented case of the vermiform appendix located in the ascending colon of a young South Asian male who presented with right upper abdominal pain. The ultrasound scan of the abdomen failed to visualise the appendix in the right iliac fossa. Persistent symptoms despite conservative therapy and elevated inflammatory markers warranted an open laparotomy. The histology further confirmed acute appendicitis.

**Clinical discussion:**

Atypical locations and congenital anomalies of the appendix are relatively rare entities. Appendicular duplication and hypoplasia are the predominant varieties of congenital anomalies. Caecal diverticula might mimic acute appendicitis despite the relative rarity and absence of all three layers of intestinal wall, which could be of use in distinguishing an abnormally located appendix.

**Conclusion:**

Such deviations from the norm lead to atypical clinical and imaging findings where operative interventions might be required in place of non-operative care, especially in instances of persistent symptomatology.

## Highlights

• Human appendix has a variable course although congenital anomalies related to its position are rare
• Unusual anatomical positions lead to atypical presentations and negative/abnormal findings in appendicular pathology.
• Hypoplasia, agenesis, duplication, triplication and diverticular formation are rare congenital anomalies of the appendix.
• Appendix located in the ascending colon has never been reported in the English literature so far.

## 1 Introduction

Acute appendicitis is the most common aetiology in patients attending emergency treatment units with lower abdominal pain [1]. Despite its rudimentary nature, the human appendix continues to baffle clinicians with varied anatomy with significant clinical and surgical implications [2]. Numerous uncommon positions of the appendix have been studied and documented extensively so far [3], [4], [5]. This is the first documented report of the vermiform appendix located in the ascending colon of a young South Asian male who underwent exploratory laparotomy due to persistent abdominal pain with raised inflammatory markers and inability to visualise the appendix with a negative ultrasonic study of the abdomen. This case report is reported in line with SCARE criteria [6].

## 2 Case description

**Fig. 1:**
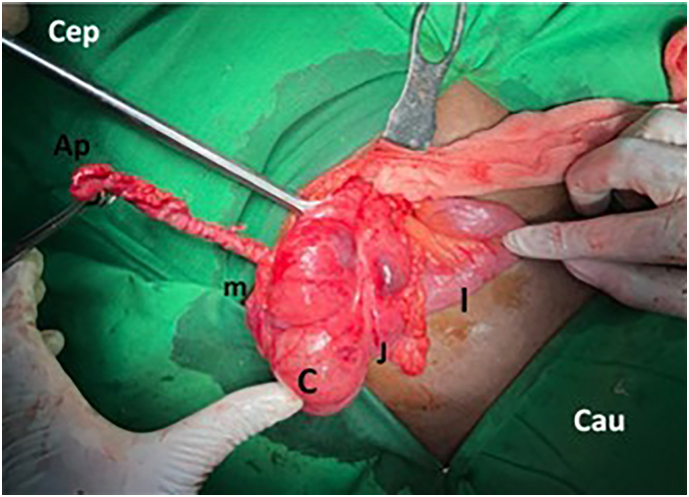
Abnormally placed appendix (Ap) in ascending colon (A). C, caecum; I, ileum; J, ileocecal junction; m, mesoappendix. Cep, cephalic; Cau, caudal.

A 28-year-old previously healthy South Asian male was admitted with fever, loss of appetite, and right upper abdominal pain for 2 days. He did not disclose any recent trauma, urinary symptoms, or altered bowel habits. On examination, there was tenderness slightly above McBurney's point with no icterus. His white cell count was elevated (15,000/mm^3^) with neutrophil predominance. C reactive protein was 80 mg/L. The urine full report was normal. Ultrasound of the abdomen did not show radiological features of acute cholecystitis, liver, or renal abnormalities. The appendix was not visualised in the right iliac fossa. An X-ray abdomen did not yield any abnormalities. Persistent symptoms despite analgesic therapy and elevated inflammatory markers prompted an open laparotomy as the laparoscope was out of order. Under general anaesthesia, a lanz incision was made by an experienced general surgeon. The appendix or remnants were not identified at the normal anatomical position. Instead, an inflamed, unruptured, 12 cm appendix was found roughly 4 cm from the ileocaecal junction at the lateral border of the ascending colon with a healthy base (Fig. 1).

**Fig. 2:**
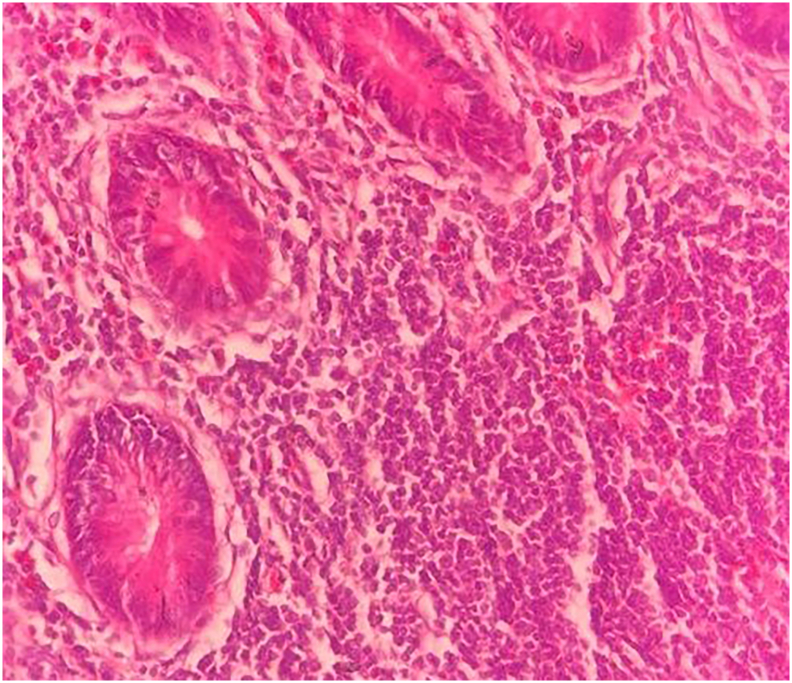
Transmural (consisting of all three layers) acute and chronic inflammation extending into the serosa in the appendix. No evidence of malignancy or diverticular formation. (H& *E*- 10 × 40).

The mesoappendix was found to have a normal origin, interposed between the terminal ileum and the caecum, containing the appendicular artery. It curled around the base of the caecum and was lengthier than usual. There was no diverticular formation, duplication, or altered positioning of the caecum or ileocaecal junction. A routine appendicectomy was performed. Histology further confirmed acute appendicitis (Fig. 2).

The patient made a full recovery with the resolution of his symptoms with antibiotic cover for 24 h postoperatively.

## 3 Discussion

The appendix is located in the posteromedial aspect or fundus of the caecum, about an inch caudal to the ileocaecal valve. Despite the majorly constant origin, it might pursue different pathways. Still, no plausible description is found in the current literature explaining this phenomenon [7]. Developmental anomalies of the appendix are extremely rare, mainly consisting of agenesis and duplication [2], [8]. Triplication, hypoplasia, diverticular formation, and intramural appendix are also described in the literature [9]. The clinical significance of such is the atypical symptomatology, the possibility of missed or misdiagnosis, false ultrasonic findings, and associated other anomalies, especially in cases of congenital abnormalities of the appendix, necessitating knowledge and a high degree of suspicion among attending clinicians. With regard to our patient, it is an extremely rare occurrence for the appendix to be located in the ascending colon. A literature review revealed cases of ectopic appendix, mobile caecum, and malrotation of the bowel with subsequent unusual positions of the appendix, which have led to atypical symptomatology and/or atypical yield of data during imaging. However, in all such instances, the relationship between the caecum and the appendix was as usual [10], [11], [12]. Another sensible explanation is hypoplasia of the appendix with an associated diverticulum of the caecum; however, the latter is uncommon and the majority of the caecal diverticula are ‘false’, denoting the absence of all three layers of the intestinal wall. Given the anatomical and histological findings in our patient and the extreme rarity of the association described earlier, we excluded it as a means of a possible explanation in this case.

Atypical presentations of acute appendicitis might mimic other abdominal pathologies with a non-exhaustive list that includes acute cholecystitis, acute pyelonephritis, diverticulitis, gastroenteritis, and ureteric colic [13], [14], [15]. The role of imaging in aiding the diagnosis and subsequent clinical decisions on management of acute appendicitis is already proven with evidence [1]. The quoted sensitivity of point-of-care ultrasonography in diagnosing acute appendicitis is around 76 %, whereas this is around 99 % for computed tomography (CT) [16]. The most up-to-date guidelines suggest repeating ultrasound studies with clinical observation or CT studies in adults with initial negative ultrasound studies [1]. Ultrasound findings could be operator dependent and affected by the anomalies, position, and course of the appendix as well [17]. CT studies are a precious commodity in the developing world and might not be immediately accessible. Persistence of atypical symptoms despite non-operative management by way of rational medical therapy and negative imaging thus suggests surgical interventions. In our patient, we had to resort to an open laparotomy due to the unavailability of a laparoscope. However, the current consensus recommends laparoscopy over open surgery [1].

## 4 Conclusion

The anatomical origin of the appendix is nearly universally constant despite its variable course and symptomatology. The most recent guidance suggests the use of clinical features supplemented with ultrasonography in the confirmation and management of acute appendicitis in place of computed tomography in every patient. Here, we reported the first case of appendix in ascending colon with atypical clinical and ultrasonic features in the absence of immediate access to CT. This extremely rare occurrence, which is most probably of congenital variety, defies any current embryological explanations, but so do our attempts at untangling the variable course of the appendix itself. It further reinforces the fact that clinical suspicion in surgical ailments has a significant role to play in decision-making and, most importantly, the outcome.

## Funding

This research did not receive any specific grant from funding agencies in the public, commercial, or not-for-profit sectors.

## Declaration of competing interest

None.
